# Glycerine of Tannin

**Published:** 1871-01

**Authors:** Meredith Clymer


### THE GLYCERINE OF TANNIN.

BY MEREDITH CLYMER, M. D.

An incorrect formula for the glycerine of tannin is given in the *Medical Record*, November 1st, p. 596. It is official in the British Pharmacopœia of 1867, and the directions for its preparation are: "Take of tannic acid one ounce; of glycerine, four fluid ounces. Rub them together in a mortar, then transfer the mixture to a porcelain dish, and apply a gentle heat, until ample solution is effected."

Dr. Sidney Ringer first published his remarks on the employment of this preparation in *The Practitioner*, July 1868. He says: "It appears to be but little known, and consequently but little used, while, in my opinion, it proves of great service in many diseases;" and he mentions ozœna; the reddish, sanious, or purulent discharging excoriations in the inside of the nose after scarlet fever or measles, with the consequent eczema of the upper lip; the thick, lumpy, greenish black, often offensive, discharges from the nose, generally chronic and intractable; the thin, sanious or purulent discharges from the ears, in weak, unhealthy children, particularly during convalesence from long illness; the early stages of eczema, when the skin is red, tumid, and weeping; the eczema which is limited to behind the ears of children; inflammations of the throat, after the acute symptoms have subsided, and particularly in those cases where there is general relaxation of the uvula and pharynx. In his Handbook of Therapeutics, just published, Dr. Ringer adds to the list of affections in which this combination does good.

Having used glycerine of tannin since the appearance of Dr. Ringer's paper, in most of the disorders just mentioned, I can bear witness to its efficiency. I have also found that in chafing (intertrigo) both in adults and children, it gives prompter relief than any other means. It will remove almost immediately the annoying itching of pruritus ani. Stuffing of the nose in syphilitic children, so characteristic and troublesome, and hindering sucking is for awhile relieved by it. In vaginal leucorrhœa it is of much and immediate service. The canal should be loosely packed with wads of cotton-wool, thoroughly moistened with it. Excoriations of the os tincæ, particularly where there is a boggy os, with a greenish yellow or sometimes sanious discharge, may be dressed with glycerine of tannin. After cleansing the parts, a cotton wad well saturated with the preparation is to be placed completely round the os and vaginal cervix, and another wad, moistened with glycerine, over this; the dressing to be changed every twenty-four hours. In chronic gonorrhœa and gleet, injections of glycerine of tannin, diluted from one-half to two-thirds, will often prove quickly curative ; it should remain in the urethra for five minutes.

Dr. Ringer writes: " In phthisis a frequent hacking cough is often dependent on the state of the throat, and can be allayed by this application. A good night's rest may be obtained by applying the paint just before going to sleep. A small quantity of morphia added to the glycerine of tannin still further increases its soothing, sedative power on the throat. The paroxysms of simple, uncomplicated whooping-cough may be most considerably lessened in frequency and violence by well sponging out the pharynx : it should be carried low down, and be brought well in contact with the epiglottis and the neighboring parts. . . . May be painted on the mucous membrane of the mouth in ulcerative stomatitis." (*A Handbook of Therapeutics*, p. 221.) In throat affections, I have not found it so efficient as other astringents, and by no means agreeable to the patient, though Dr. R. says, " It has the further advantage of not possessing a bad taste."—*Half-Yearly Abstract.*

